# Experimental Research on Sheared Edge Formation in the Shear-Slitting of Grain-Oriented Electrical Steel Workpieces

**DOI:** 10.3390/ma15248824

**Published:** 2022-12-10

**Authors:** Łukasz Bohdal, Agnieszka Kułakowska, Radosław Patyk, Marcin Kułakowski, Monika Szada-Borzyszkowska, Kamil Banaszek

**Affiliations:** Department of Mechanical Engineering, Koszalin University of Technology, Racławicka 15–17 Street, 75-620 Koszalin, Poland

**Keywords:** electrical steel, shear-slitting, sheared edge, magnetic properties, optimization

## Abstract

This study sought to experimentally develop guidelines for shaping 0.3-mm-thick cold-rolled grain-oriented ET 110-30LS steel using a shear-slitting operation. Coated and non-coated steel was used for the analysis. The coated sheet had a thin inorganic C-5 coating on both sides applied to the C-2 substrate. The first part of this paper presents an analysis of the quality of the cut surface depending on the adopted machining parameters, which were the control variables on the production lines. The second part presents an analysis of the magnetic parameters of the cut samples, which allowed for the specific impact of the quality of the cut edge on the selected magnetic features. Finally, an optimization task was developed to obtain a set of acceptable solutions on the plane of controllable process variables such as slitting speed and horizontal clearance. The obtained results can be used to control the shear-slitting process on production lines and obtain high-quality workpieces.

## 1. Introduction

The dynamic development of manufacturing techniques is related to the ever-increasing requirements for product quality, manufacturing operations reduction, and the assurance of high efficiency in the machining process. Accordingly, there are many difficulties involved in the correct development and proper implementation of technological processes that meet these requirements. Modern materials with promising applications are appearing on the market, but to realize their potential, they will require continuous development and investigation into the the possibilities of their processing and shaping [1,2]. The trend of continuous miniaturization in electronic and magnetic components also poses new challenges to production processes. In many cases, at the production stage, it is necessary to change the geometry and surface properties of the workpiece material by separating its fragments or applying mechanical pressure. Therefore, mechanical cutting processes are constantly used in various industries, such as the electromechanical, electrotechnical, automotive, food, and paper industries. In the electrotechnical industry, a notable challenge is to ensure not only the appropriate mechanical properties, but also the magnetic properties of the shaped products. The optimization of various methods of cutting facilitates the manufacture of a product of high technological quality in one operation. The products must have no burrs and minimum shape deviations and the deformation affected zone must be of minimum width [3,4,5]. In the electrotechnical industry, electrical steels and amorphous and nanocrystalline tapes are the basic magnetic materials used, among others, for the production of electrical machines such as electric motors, transformer cores, and generators [6,7,8]. Despite the availability of models that allow for forecasting the technological quality of products in the mechanical cutting process in terms of the state of the cut surface, the proper selection of technological parameters for the process is complicated, especially in the case of shaping magnetic materials. This is due to the lack of data in the literature regarding the settings of machining parameters, for example, for the shear-slitting of grain-oriented silicon electrical sheets in the context of final cutting surface quality and the obtained magnetic properties of the product. Thus, there are known problems that occur in the manufacture of electrical machinery such as transformers and electric motor cores.

In the case of electrical steel cutting, the difficulty in production lines is in ensuring appropriate quality in the cut edge with minimal interference with the magnetic characteristics. It is necessary to analyze the physical phenomena occurring in the contact zone of the tools with the material that is shaped during the process. Knowledge of these phenomena is crucial for proper process control and the design of new tools. Creation of built-up edges on tools, process instability, changes in physicochemical properties in the cutting zone, and low dimensional and shape accuracy of the product are typical problems on production lines related to magnetic material processing that uses cutting techniques [9,10]. The occurrence of slivers, burrs, microcracks in the material, and edge bends are the reasons for poor-quality cut surfaces and result in the generation of waste [11,12].

Previous studies have discussed the selection of an appropriate cutting technology (punching, abrasive water jet cutting, laser cutting) for shaping these steels [13,14]. There is a lack of data on the correct conditions for the implementation of mechanical cutting processes to obtain appropriate cut-surface quality and the least possible interference in the magnetic properties of electrical materials. However, there are no universal guidelines for process control to obtain high-quality products. The technological quality of electrical steel products is determined not only by the characteristic features of the sheared edge, but also by the magnetic properties, which are often degraded as a result of incorrect process performance. In two previous papers [15,16], the authors conducted research on the influence of cutting speed on the magnetic properties of amorphous tapes. The hysteresis losses were significantly influenced by the increased deformation zones of the material, which were caused by the excessive wear of the cutting tool and the formation of burrs on the intersection surface. Another paper [17] presents an analysis of the process of fine punching, which helps to ensure a higher quality in the cut surface than the standard process of punching electrical sheets due to the greater triaxial compressive stress in the cutting zone caused by the use of a wedge root and a small cutting clearance. In addition, some researchers [18] developed the FEM model to facilitate the determination of the optimal clearance for punching electrical sheets with non-oriented grain. The modeling results were experimentally verified for each clearance value obtained.

Some major challenges lie in the correct control of the cutting speed, the value of the cutting clearance, and the appropriate selection of the geometry of the cutting tools depending on the type of material being cut for obtaining a product of appropriate quality. The problem of wear in cutting tools during the forming of electrical sheets has also been analyzed [13,16,17,18,19,20].

It has been shown that the magnetic properties are mostly influenced, apart from concentrated stresses in the cutting zone, by grain deformation and the crystallographic structure, which depend especially on the amount of cutting clearance and speed [21]. In several papers [13,15,16], attempts were made to determine the effect of cutting speed on changes in magnetic properties. The authors of two other papers [16,22] made an attempt to investigate the influence of cutting tool wear and cutting process speed on the width of the deformation zone and the magnetic properties of electrical sheets. Local changes in the cutting edge geometry due to wear resulted in the appearance of burrs on the cut surface, an increased deformation zone, and hysteresis losses. In another two papers [13,23], hysteresis losses also increased as a result of changes in the microstructure of materials such as dislocations or stresses. The test results also showed that sharp tools caused less magnetic degradation than worn, blunt tools. In one study [24], the quality of the cut edge and negative changes in magnetic parameters were significantly influenced, apart from the processing parameters, by the methods of attaching metal sheets to punching dies. Variable boundary conditions were particularly visible in the cutting process on circular shears where the sheet metal exhibited longitudinal movement.

In shear-slitting, the material separation mechanism is often very difficult to control because the tools rotate and the material is not rigidly held [25,26]. If appropriate process conditions are not ensured, excessive tensile stresses may concentrate in the cutting zone, causing local tearing of the material. Two papers [27,28] analyzed the influence of the cutting process on selected magnetic properties of grain-oriented electrical steel cut from sheets with a width of 40 to 660 mm. A significant influence of the construction of tools on the mechanism of material deformation in the cutting zone was found, and the influence of the process conditions on the iron loss of steel was analyzed. Hubert et al. [29], using FE modeling, analyzed the stress distributions in the shearing area under the use of two rotary tools. Particular attention was paid to building an effective process model and appropriate FE discretization in the areas of contact between tools and material. It has been shown that the cutting clearance is an important factor influencing the stress values. The significance of the value of the knife rake angle on the formation of burrs on the cut surface in the case of metal alloys has also been demonstrated [30]. By controlling the rake angle, it is possible to increase the quality of the cut part of the sheet [31]. In the case of electrical sheets with a small thickness, t = 0.1–0.3 mm, the rake angle control alone is not sufficient, because its selection depends on the cutting speed.

The objective of this study was to research the impact of main shear-slitting technological process parameters on the cut surface formation mechanisms and the final quality of the sheared edge of grain-oriented electric steel ET 110-30LS. In the process of cutting electric steels, the selection of cutting parameters is often done by trial and error, which extends the duration of the process and the amount of waste. In the case of defects on the cut edge, smoothing and deburring operations are used, which increase production costs. Currently, the literature and industry lack information on how to minimize inter-laminar short-circuit faults between the laminations of electrical machines, which are two of the main challenges for suppliers and customers of electrical steels. In several papers [32,33,34], additional power losses caused by interlayer faults depend on many factors, including the location of the fault points. One of the reasons for the formation of short-circuit points is the formation of burrs on the edges of packaged sheets and the deviation of the sheet shape. In many cases, it is necessary to use deburring operations. In some critical applications, e.g., large rotating machines, a second coating layer is applied to the laminates after the punching or cutting deburring operation. However, deburring processes are usually not very thorough and can reduce the accuracy of machined parts, damage the edges or surface of the sheet, and create additional stresses in the lamination. In addition, the deburring process requires an additional machining step and additional production time, leading to additional costs. Partial interlayer failure in the stator cores of rotating machines can be more destructive than transformer cores because the layers of the stator cores are welded or held together by key bars or a casing at the base of the stator yoke.

Therefore, it is crucial to define guidelines for the proper control of the cutting process so that at the stage of its duration, the correct condition of the cut edge without defects is already obtained without interfering with the magnetic properties. The novelties of our study include the following:

- Determination of appropriate conditions for the shear-slitting process to obtain minimum burr heights and minimum shape deviations, enabling the correct packaging of sheets. Currently, there is no information on how to select process parameters and conditions to minimize these features at the same time. Knowledge on this subject will allow for the manufacture of products ready for direct packaging without the need for additional machining operations.
- Comprehensive tests for coated and uncoated sheets. Electrical engineering materials in industry are shaped by cutting techniques in different phases, often before or after coating. Currently, the literature lacks information on how to cut such materials depending on their condition. Here, we have proposed universal solutions that can be used for the cutting process of both coated and uncoated materials.
- Optimization techniques that allow for determining the areas of acceptable solutions and the optimal solution. Based on our results, these techniques can be used on production lines, enabling technologists to select appropriate conditions for the cutting process, depending on the assumptions made.
- Partial experimental research, result analysis, and multi-criteria optimization of the cutting process, which enables the development of an effective numerical model of the process for blanking parts for electric machines. On the basis of the obtained research results, a computer model was developed using the finite element method for the process of blanking parts from grain-oriented electrical sheets for the construction of an electric transformer. The developed model is aimed at verifying the results of fragmentary tests and implementing research conclusions in production.

Due to the complexity of the problem, the research was carried out in several stages. In the first stage, research was carried out on the influence of the most important technological parameters of the process on the quality of the cut edge for both sheets with an *electric insulation coating* and without the coating. This enabled observation of the geometric structure of the sheared edge and analysis of the causes of its defects in the form of: burrs, bends and rounded edges, and coating delamination. Then, selected magnetic properties of the sheet metal were tested after the cutting process for the adopted processing parameters. As a result, it was possible to determine the values of the technological parameters that could most often be controlled on production lines during the process, ensuring the highest quality of the cut edge while maintaining the appropriate magnetic properties. In the final part of the paper, we present a computer model that uses the FEA of the blanking grain-oriented electrical sheet metal process for the construction of an electric transformer. The research results were implemented in industry.

## 2. Materials and Methods

### 2.1. Material Characteristics

The tests were carried out on cold-rolled grain-oriented ET 110-30LS steel of 0.3 mm thickness. The choice of material was based on technological conditions. In the case of shaping electrical sheets, there are no guidelines for the proper control of the cutting process. A thickness of t = 0.3 mm was chosen due to this thickness having the greatest applicability for the construction of electrical devices. The material, despite the quality guarantees provided by the supplier, was subjected to the following laboratory tests: hardness and mechanical properties. The material we analyzed is used in the construction of power and distribution transformer cores and in the production of current transformers, voltage transformers, medium and large high-efficiency generators, reactors, magnetic screens, and coiled audio transformer cores. Coated and non-coated steel was used for the analysis. The coated sheet had a thin inorganic C-5 coating on both sides, applied to the C-2 substrate (labeled in accordance with ASTM A976-13: 2018 [35]). The C-5 coating has a very good electrical insulating quality, was 1.5–3.0 µm thick per side, and had good adhesion to the substrate. This ensured insulation resistance of >15 Ω cm^2^, and the coating is also resistant to annealing up to 840 °C in a non-oxidizing atmosphere. Coated and uncoated sheets were selected to develop guidelines for the implementation of the process for two material variants. This choice also allowed for clarification on whether universal settings of the process parameters for these materials could be used or whether they needed to be modified to maintain appropriate quality in the workpiece.

The material hardness was tested using the Brinell and Vickers methods. Measurements were made with HPO-300 and PTM-300M hardness testers. The basic mechanical properties of the material were determined by carrying out a material tensile test on a Zwick/Roell Z400 testing machine. The samples were made in accordance with the PN-EN 10002-1 + AC standard [36]. Ten replications of the test were used for each material. The results are summarized in Table 1 and Table 2.

### 2.2. Experimental Setup

**Figure 1:**
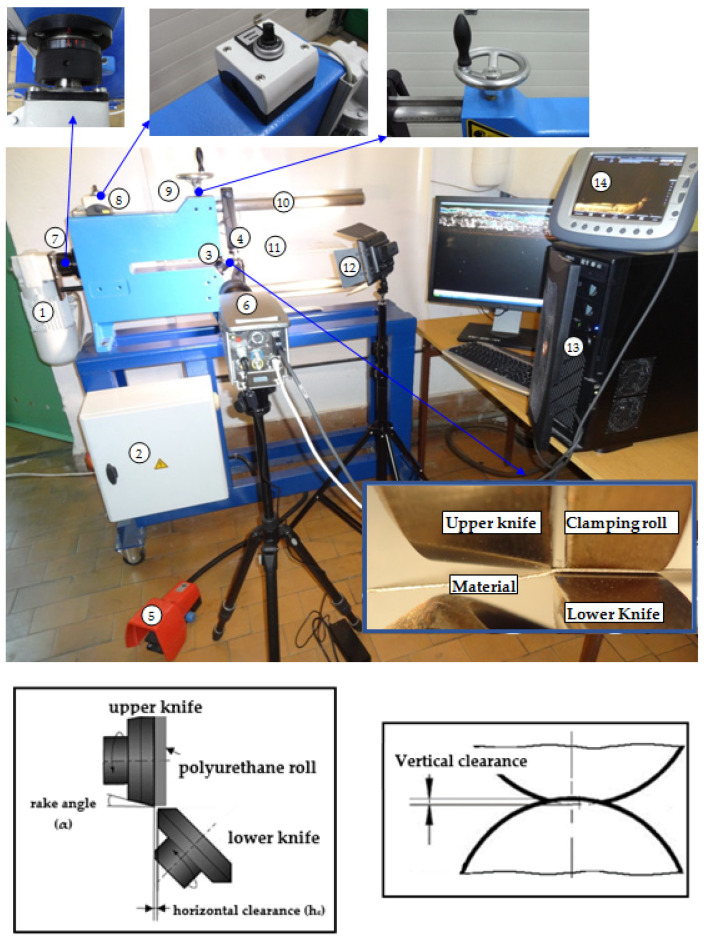
Experimental equipment and characteristic parameter definitions: 1—engine, 2—electrical system, 3—circular knives, 4—sheet stabilizer, 5—drive pedal, 6—high-speed camera, 7—threaded socket with a scale for adjusting the clearance, 8—cutting speed regulator, 9—knife overlap regulator, 10—scale for determining the diameter of cut discs for curvilinear contours, 11—sheet metal, 12—LED lamp, 13—PC for archiving measurement data, 14—auxiliary screen for data recording and analysis.

To conduct experimental research, a test stand was designed that consisted of a shear-slitting machine (Prinzing Maschinenbau) and high-speed camera i-SPEED TR (iX Cameras) that was used to record the cutting process. The sheets of metal were fixed in the device with special mounts with sheet holders. Then, the machining parameters were set in accordance with the five-level rotatable experiment plan. Specially purchased additional components enabled the use of high cutting speeds, precise settings for the cutting clearance, and knife overlap (Figure 1). The machine was driven in one-stage, two-stage, or stepless mode through a motor with a gear and a brake. The drive was transmitted to the upper knife and the pressure roller made of polyurethane. The horizontal clearance *h_c_* was adjusted by a threaded socket with a scale. The slitting velocity *v* was set by a knob with a scale. Five replicates were performed for each level of the study plan.

As a result of the preliminary research, the most important factors were determined. The input factors included horizontal clearance (*h_c_*) and cutting velocity (*v*). The values of the process parameters are summarized in Table 3.

**Figure 2:**
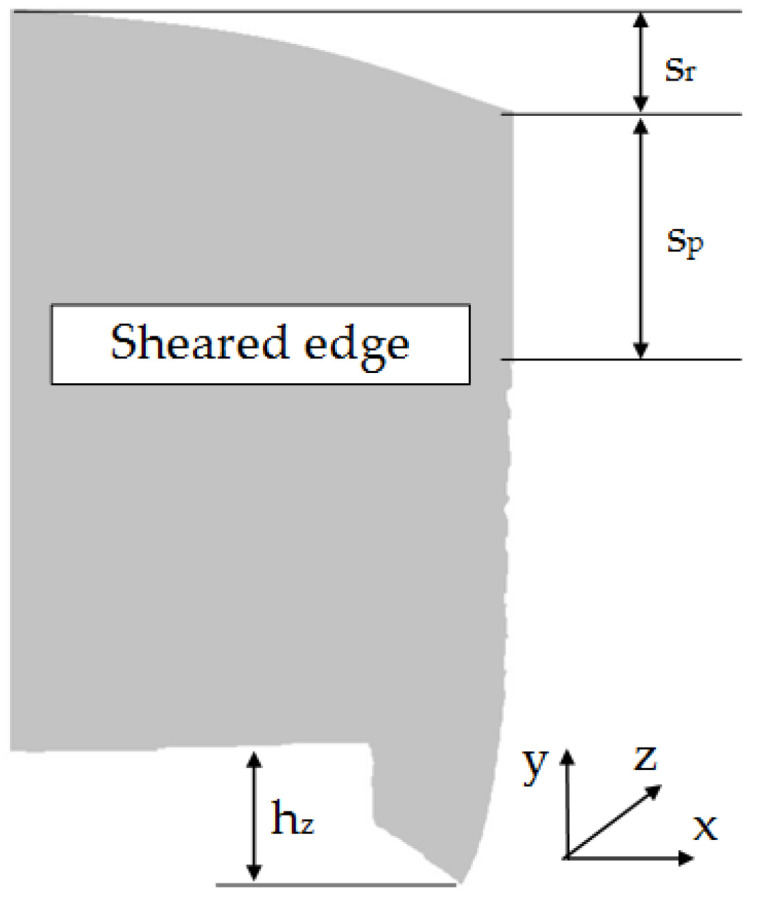
Typical sheared edge contour with characteristic areas, cross-sectional scheme; *s_r_*—roll-over, *s_p_*—sheared-burnished, *h_z_*—burr.

After the cutting process, the characteristic features of the cut edge were measured, including the height of the burr, the width of the roll-over, and the width of the sheared-burnished area (Figure 2). Measurements were taken in random places along the cutting line using a measuring microscope (Kestler-Vision Engineering Dynascope) with the ND 1300 Quadra-Chek measuring system. The LEXT OLS4000 confocal laser microscope from OLYMPUS was used to measure the geometric structure of the tested steel’s cut edge after cutting. This microscope made it possible to obtain images with excellent quality and accurate 3D measurements using the advanced UIS2 Optical System (infinity correction, non-destructive method). The professional TalyMap Platinum software (version 7.4) was used to analyze the measurement results. Using the data obtained from the measurements of microtopography, the program made it possible to determine the value of the parameters of the geometric structure of the surface of ET 110-30LS steel after cutting, facilitating a graphical presentation of the geometric shape of the measured areas and their profiles.

## 3. Experimental Results and Discussion

### 3.1. Sheared-Edge Topography

Sample photos of the sheared edge and its surface topography maps are shown in Figure 3, Figure 4, Figure 5, Figure 6, Figure 7, Figure 8, Figure 9 and Figure 10.

In the case of mechanical cutting processes, irregularities on the sheared surface emerge after the process in the macro and micro scales, the height and arrangement of which may affect the tribological properties of the steel after the cutting process [3,5]. The type of breakthrough may have a large impact on the operational properties of machine elements through the frictional conditions on the contact surfaces, contact stress, joint tightness, fatigue strength, or magnetic properties. Production deficiencies in surface preparation may cause mechanical damage, e.g., fatigue cracks. To improve the fatigue strength of details, rolling and burnishing processes are used. In one study [37], the authors presented a theoretical and experimental analysis of the roller-burnishing technique to achieve isotropic surface topography on cylindrical components made of austempered ductile iron (ADI) casting. Their results showed that their proposed solutions greatly improved surface roughness and eliminated the kinematic-driven roughness pattern of turning, leading to a more isotropic finishing that can reduce the negative impact of mechanical cutting on fatigue cracks.

**Figure 3:**
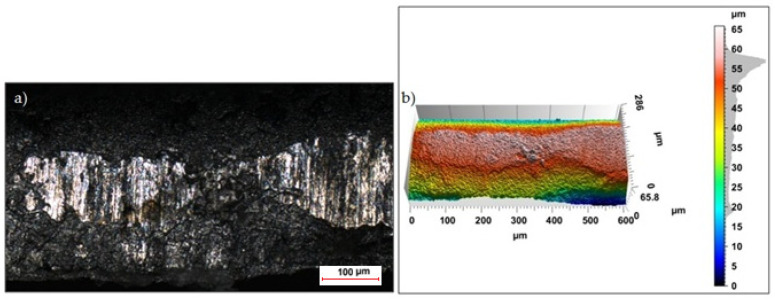
Views of the surface of the cut edge (*h_c_* = 0.04 mm and *v* = 10.2 m/min): (**a**) surface photography, (**b**) surface topography (uncoated steel).

**Figure 4:**
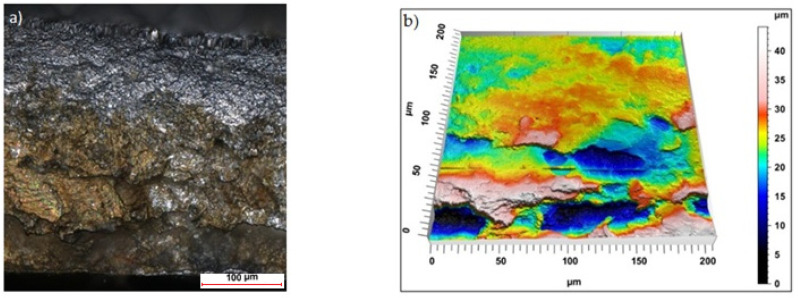
Views of the surface of the cut edge (*h_c_* = 0.04 mm and *v* = 10.2 m/min): (**a**) surface photography, (**b**) surface topography (coated steel).

Specimens cut with a clearance of *h_c_* = 0.04 mm and cutting speed *v* = 10.2 m/min for uncoated material were characterized by a clear sheared burnished transition into fracture area in the separating material (Figure 3). The width of the sheared burnished area was greater than the width of the fractured area. The material was characterized by a slight edge-rounding and perpendicular deviation, which did not significantly reduce the quality of the cut edge. This indicates the occurrence of tensile material in selected areas, along with shear in the gap between the cutting edges, which caused local changes in the width of the sheared burnished and its unevenness. The insulating coating influenced the topography of the cut surface. Compared to the uncoated material with clearance of *h_c_* = 0.04 mm and speed of *v* = 10.2 m/min, the samples were characterized by a smaller rollover in the sheared surface and smaller deviations in perpendicularity (Figure 4). The sheared burnished area was mostly dull with local shiny areas. A significant share of tensile stresses was visible in the areas of the fracture formation, indicated by the size and number of pits with a depth of more than 30 µm. On the edge of the burr, in some areas along the cutting line, fragments of the electrically insulating coating, which is a form of built-up edge on the cut surface, were visible.

**Figure 5:**
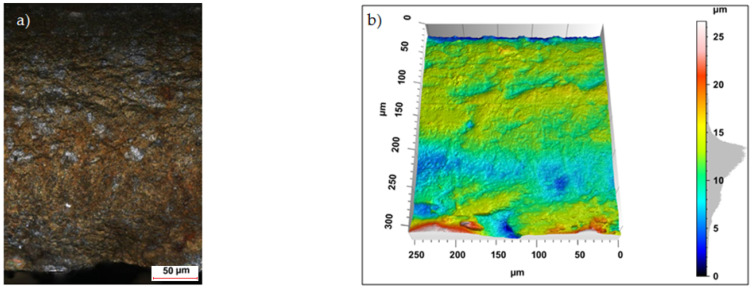
Views of the surface of the cut edge (*h_c_* = 0.08 mm and *v* = 10.2 m/min): (**a**) surface photography, (**b**) surface topography (uncoated steel).

Increasing the cutting clearance increased the degree of roll-over of the cut edge of the product. In the case of cutting with clearance of *h_c_* = 0.08 mm and speed of *v* = 10.2 m/min the samples were characterized by a matt sheared burnished without a clear transition border in the fractured region (Figure 5).

**Figure 6:**
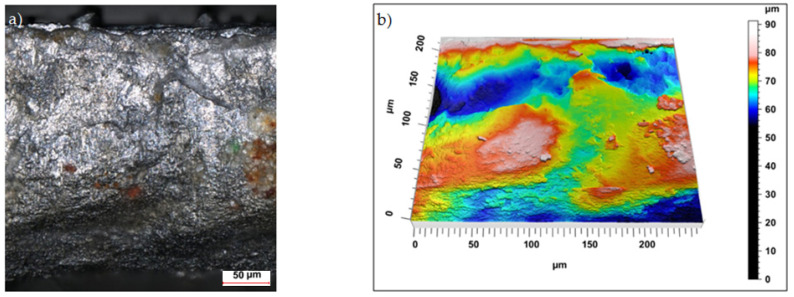
Views of the surface of the cut edge (*h_c_* = 0.08 mm and *v* = 10.2 m/min): (**a**) surface photography, (**b**) surface topography (coated steel).

The increase in clearance for coated material increased the area of deformation concentration and enlarged the area of the built-up edge, which also includes the coating, which was deformed and curved towards the center of the cross-section (Figure 6).

For this case, zones of irregular sliding fracture were also visible, where the arrangement and depth of the furrows indicated the occurrence of alternating phases of flow or cracking along the cut line already at the beginning of the process.

**Figure 7:**
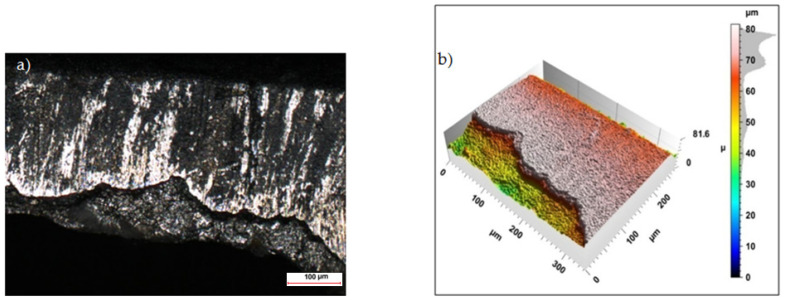
Views of the surface of the cut edge (*h_c_* = 0.02 mm and *v* = 17.5 m/min): (**a**) surface photography, (**b**) surface topography (uncoated steel).

**Figure 8:**
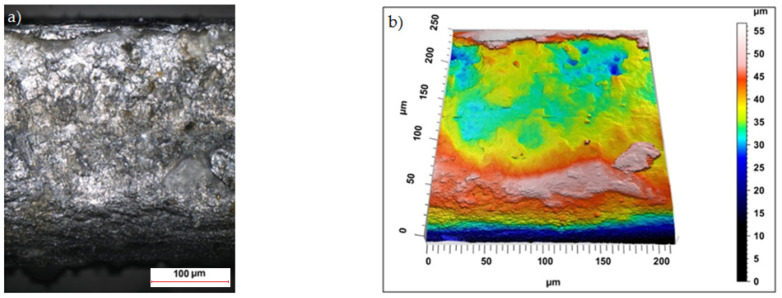
The view of the surface of the cut edge (*h_c_* = 0.02 mm and *v* = 17.5 m/min): (**a**) surface photography, (**b**) surface topography (coated steel).

For cutting with minimum horizontal clearance of *h_c_* = 0.02 mm and cutting speed of *v* = 17.5 m/min the samples had a visible transition boundary of the sheared burnished area in the fractured area (Figure 7). The width of the sheared burnished area, as in the case of cutting with clearance of *h_c_* = 0.04 mm and speed of *v* = 10.2 m/min was greater than the width of the fracture area. Coated steel samples had a shiny sheared burnished area with matte areas and local depressions with a maximum depth of approximately 10 µm (Figure 8). The rounding of the cut edge and the built-up layer of the insulation coating overlapping the cut surface can be seen.

**Figure 9:**
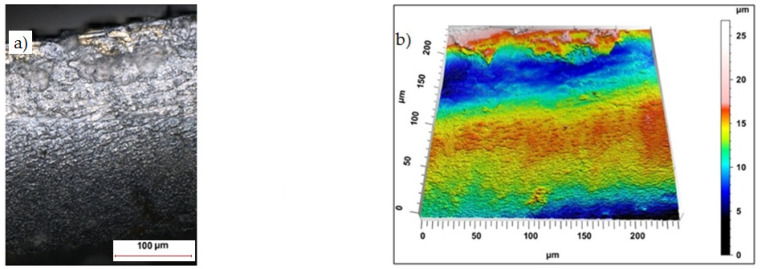
Views of the surface of the cut edge (*h_c_* = 0.06 mm and *v* = 17.5 m/min): (**a**) surface photography, (**b**) surface topography (uncoated steel).

**Figure 10:**
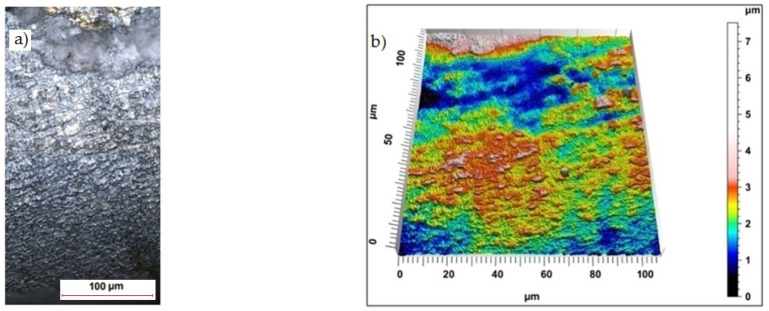
Views of the surface of the cut edge (*h_c_* = 0.06 mm and *v* = 17.5 m/min): (**a**) surface photography, (**b**) surface topography (coated steel).

Increasing the clearance value from *h_c_* = 0.02 mm to *h*_c_ = 0.06 mm with a cutting speed of *v* = 17.5 m/min resulted in a slight increase in the regularity of the sheared burnished area, which was still very smooth for uncoated material (Figure 9). The structure was slightly grainy but without the presence of peaks, which reached their maximum values in the area of the burr, creating local surface irregularities in the direction perpendicular to the cut surface. However, the burr height was minimal for both cases. The increased flow of eddy currents was caused by burrs, which contribute to increased losses due to additional electrical paths [33]. In the case of the coated material, the increase in horizontal clearance resulted in the elongation of the plastic flow phase and the emergence of a wide sheared burnished zone with a homogeneous structure and the minimum peak height. The breakthrough was grainy and shiny. A slight roll-over of the edge was visible (Figure 10).

### 3.2. Sheared-Edge Quality

The quality of the sheared edge after the mechanical cutting process is usually analyzed by measuring the width of characteristic areas (Figure 2) on the cut-edge profile and burr height *h_z_*. From a technological point of view, it is very important to reduce the burr height below 20% of the thickness of the material being cut. The sheared burnished zone width should be maximized and roll-over minimized. Currently, there are no guidelines regarding the optimal values of these zones in terms of product quality nor an analysis of their influence on the magnetic properties. In a later part of the study, we analyzed the influence of the tested parameters on the selected magnetic properties of the material. The analysis of the test results showed a strong influence of cutting speed and clearance on the formation of the width of individual zones on the cut surface, both in the case of sheets with and without an insulating coating.

**Figure 11:**
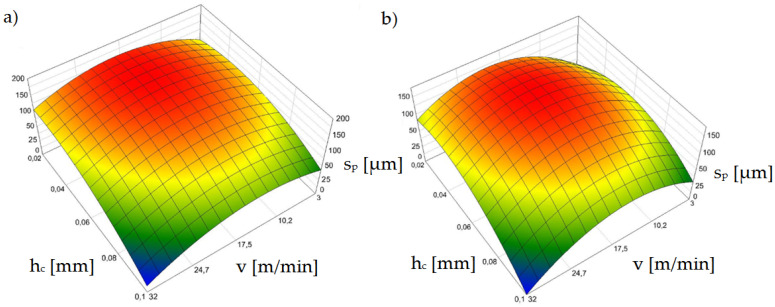
Graph of the dependence of the width of the sheared burnished zone (*s_p_*) on the cutting speed (*v*) and the horizontal clearance (*h_c_*): (**a**) uncoated material, (**b**) coated material.

Figure 11 shows the effect of horizontal clearance and slitting speed on the width of the sheared-burnished zone *s_p_* for uncoated and coated material.

The settings of the input parameters for which the zone of the sheared burnished area is the largest represent conditions for a proven steady state for the process, as confirmed in prior research [38,39,40]. In punching and blanking processes, ensuring process stability is easier than in shear slitting because the material is not displaced during the process. Therefore, the material is less exposed to local large increases in tensile stress in the cutting zone and tearing, causing an increase in the fracture areas and burr. A very important issue is the correct selection of the processing speed, which would ensure the high efficiency and appropriate technological quality of the process. In one paper [41], a mechanistic model for cutting force prediction was presented. A series of cutting trials using austenitic stainless steels and high process speeds were conducted to obtain expressions of the proper force factors. Expressions were developed for individual shear coefficients, taking into account the variable depths of cut and process speed. Deformation and strain-hardening of austenitic stainless steels at high cutting speeds were discussed. Another paper [42] presents a complete analysis of the principal beneficial aspects of mechanical surface treatment produced by the application of ball-burnishing at high speed. The use of such a treatment makes it possible to increase the fatigue strength of sheets.

**Figure 12:**
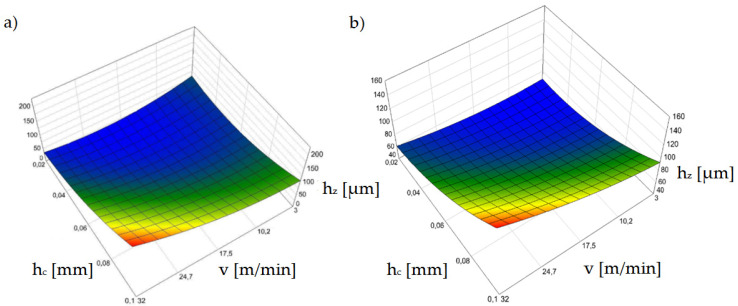
Graph of the dependence of the burr height (*h_z_*) on the cutting speed (*v*) and the horizontal clearance (*h_c_*): (**a**) uncoated material, (**b**) coated material.

Our test results for shear-slitting indicate that the lowest process stability is obtained at high cutting speeds above *v* = 24 m/min (Figure 11a,b). This especially applies to variants of horizontal clearance above *h_c_* = 0.08 mm. According to other studies [30,31,38] this is probably due to the instability of the fracture process which, at high speeds, can occur more quickly even with minimal horizontal clearances in electrotechnical materials. This increases the width of the fractured zone on the cut edge. Using clearances in the range of *h_c_* = 0.04–0.08 mm, the greatest width of the sheared burnished zone can be obtained; however, for clearances within *h_c_* = 0.08 mm, the burr increases (Figure 12). For the *v* = 3 m/min cutting speed, there was a sheared burnished zone even for a clearance of *h_c_* = 0.1 mm in the case of uncoated sheets (Figure 11a). For the coated material, when a clearance of *h_c_* = 0.02 mm was used, it was advantageous to use a cutting speed above *v* = 10 m/min. (Figure 11b).

Figure 12a,b shows the influence of the analyzed parameters on the burr height on the cut edge. Significant problems occurred when the burrs electrically connected several layers, because losses increased and there was a high risk of melting the core [33]. The use of clearances above *h_c_* = 0.06 mm caused a significant increase in the burr height for both coated and uncoated material. For clearances above *h_c_* = 0.08 mm, the burr height was too high and may cause a problem on the production line for each analyzed cutting speed. An increase in the burr contributes to an increase in the roundness of the edge and high roll-over. The use of a clearance value below *h_c_* = 0.06 mm allows the use of significant cutting speeds, even up to *v* = 24 m/min, while still obtaining an acceptable burr height value. For minimum clearance values of *h_c_* = 0.02–0.04 mm, the width of the sheared burnished area increases, which results in a significant reduction of burrs for each speed. When using a clearance of *h_c_* = 0.04 mm, it is more advantageous to use a cutting speed in the range of *v* = 3 ÷ 20 m/min for coated material and *v* = 3–28 m/min, so that the burr height does not exceed *h_z_* = 60 µm.

**Figure 13:**
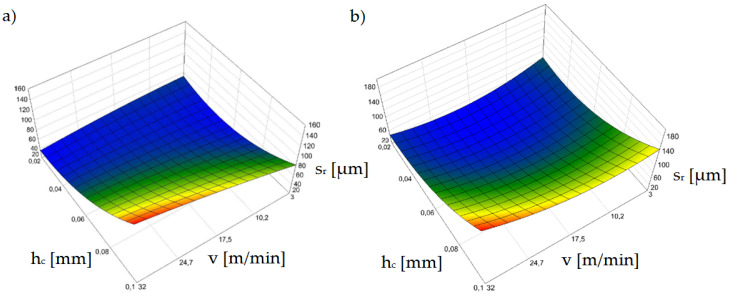
Graph of the dependence of the roll-over width (*s_r_*) on the cutting speed (*v*) and the horizontal clearance (*h_c_*): (**a**) uncoated material, (**b**) coated material.

According to a number of papers [38,43,44,45] in which the authors analyzed the processes of punching and blanking metal materials, the width of the roll-over zone depends mainly on the cutting clearance. One of these papers [44] presented the results of the analysis of the impact of the number of punch cycles on the height of burrs on the cut edge and roll-over formation for three values of the clearance of the blank at 5%, 10%, and 15% of the sheet thickness. It was shown that the intensity of the degradation of the working surfaces and punching edges of the punch were influenced by the friction path. However, our results indicate that it is also important to properly select the cutting speed depending on the adopted cutting clearance (Figure 13a,b). Excessive roll-over concentrates plastic strains over a larger area and contributes to the degradation of magnetic properties [45,46]. The use of cutting clearances over *h_c_* = 0.08 mm was found to have an adverse affect.

In this area, the effect of the insulating coating on the width of the roll-over zone is significantly increased. For coated material, the maximum roll-over values were greater than for uncoated material for each speed value. The greatest differences occurred for the cutting speed of *v* = 3 m/min when the clearance was *h_c_* = 0.1 mm. We noted that as clearances increased, ther was an excessive increase in the bending moment and local edge damage. This is related to the excessive concentration of deformations and the plastically strengthened area in the vicinity of the cut edge, as confirmed by the tests carried out in prior work [47]. The movement of defects or irregularities in the crystal, called dislocations, causes plastic deformation. This impedes the movement of the domain walls and increases hysteresis loss [47]. The use of an insulating coating results in a narrowing of the ranges of favorable parameter settings and an increase in roll-over when using minimal clearances and increased cutting speeds.

### 3.3. Magnetic Characteristics

**Figure 14:**
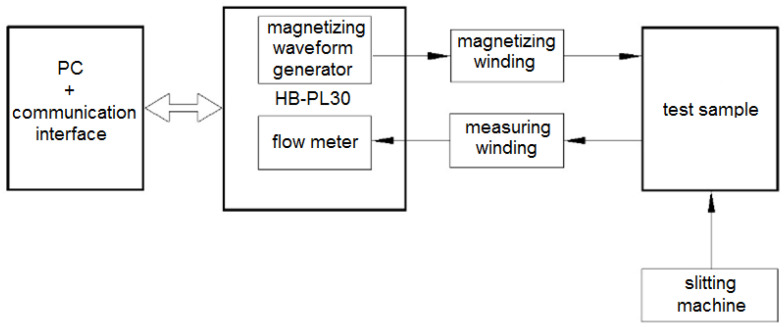
Block diagram of the test stand for testing magnetic characteristics.

There are many aspects that may have a negative impact on the magnetic properties of electrical sheets, which include excessive concentration of maximum stresses and deformations in the area of the cut edge, a wide deformation zone or the formation of burrs, built-up edges on the cutting edge, and damage to the insulating coating [31,47,48]. It was therefore important to determine to what extent the investigated process conditions affected the magnetic properties. Specially prepared samples made of laser electrotechnical steel ET 110-30LS with an insulating coating applied (closed ring samples, where outer diameter *D* = 120 mm, inner diameter *d* = 90 mm) were used for the tests. The preparation of samples was carried out according to previously described standards and procedures [46,49]. A magnetizing winding and a measuring winding were wound on each of the samples, each winding being wound uniformly to create a closed magnetic circuit and avoid magnetic flux dispersion in the material. The tests were carried out on a test stand consisting of the components shown in Figure 14.

**Figure 15:**
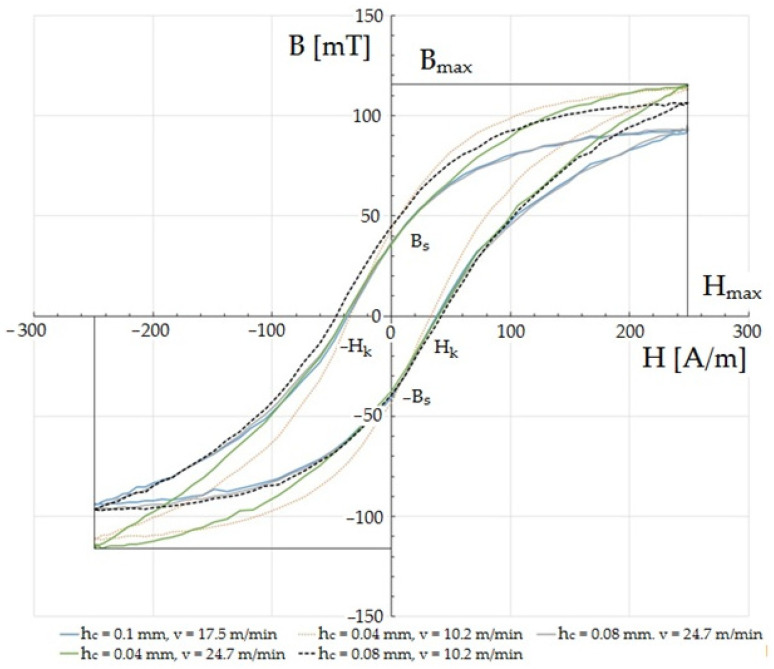
Influence of selected values of input factors of the cutting process on the magnetic characteristics B (H) of the ET 110-30LS sheet.

**Figure 16:**
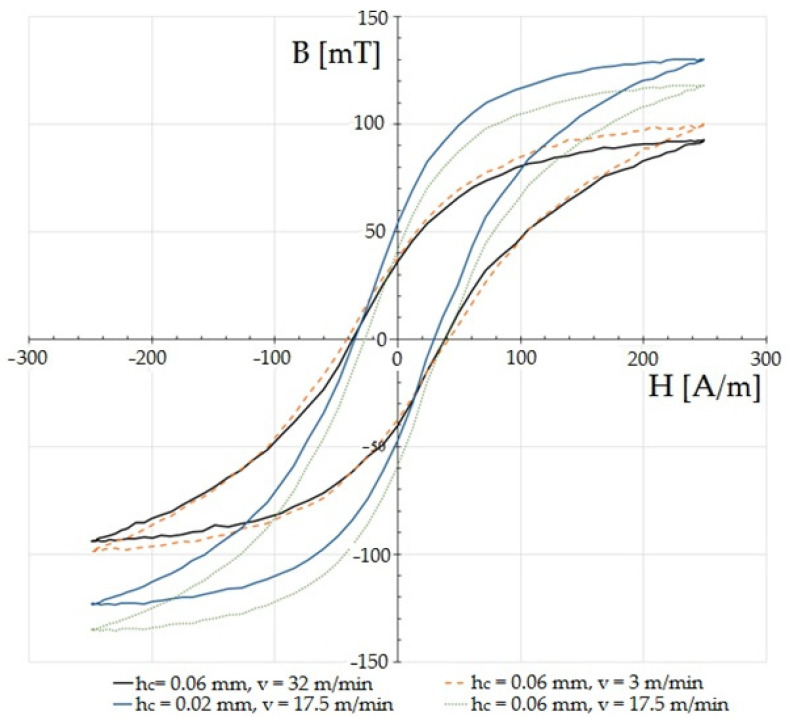
Influence of selected values of input factors of the cutting process on the magnetic characteristics B (H) of the ET 110-30LS sheet.

The magnetic characteristics were measured for the determined values of the amplitude of the magnetic field strength at *Hm* = 250 A/m. The frequency of the demagnetizing waveform was 10 Hz. Figure 15 and Figure 16 show the effect of the cutting speed and the clearance between the cutting tools on the hysteresis loops of ET 110-30LS steel with a thickness of *t* = 0.3 mm. For the tests, the rake angle of the cutting edge of the upper knife was *α* = 7°, and the vertical clearance of the knives was *c_v_* = 0.1 mm. The intensity of *H_max_* and the induction of *B_max_* are called saturation intensity and induction, respectively. *B_s_* is the remanence induction. The strength of the magnetic field *H_k_* is called the coercive force. In devices with multiple magnetization (e.g., transformer cores), hysteresis is seen as a problem because its surface area is proportional to the energy loss during one re-magnetization cycle. Given the appropriate thermal and plastic treatments and chemical composition, it is possible to minimize its surface and reduce its coercivity. In the case of soft magnetic materials, even a low range of deformation (1–10%) affects the magnetic properties [30,47,48]. In one paper [49], the authors used strength tests to show that the magnetization at saturation and remanence decrease with increases in deformation. Magnetic saturation refers to a state in which the increase of an applied external magnetic field does not increase the magnetization of the material. Remanence, or residual magnetization, is the value of the magnetic induction that remains after the removal of the external magnetic field that was magnetizing a given material. 

Among cutting processes, the greatest changes in the parameters of the hysteresis loop were shown in one paper to be caused by laser cutting [50]. As a result of high stresses and thermal deformations in the cutting zone, the hysteresis loops were strongly distorted and exhibited the largest internal surface area, i.e., total losses: the results for individual experiments showed that the shear-slitting process conditions had a significant influence on changes in the characteristic features of the magnetic hysteresis loop. Here, the negative impact of increased cutting clearance at high speeds on the characteristics of the hysteresis loop was particularly visible (Figure 15 and Figure 16). Clear changes in the shapes of the hysteresis loop could be observed in the upper-bend areas of the characteristics and saturation. When using reduced cutting clearances outside of the range of *h_c_* = 0.02–0.04 mm, the cutting speed mainly affected the characteristics of the saturation area and maximum induction (Figure 15). The parameters of the coercivity intensity and remanence induction changed slightly only for a cutting speed of *v* = 10.2 m/min in this range of cutting clearances. The effect of saturation induction decrease above clearance values of *h_c_* = 0.08 mm was particularly visible.

The increase in the intensity of coercivity and remanence induction is probably due to the increased deformation zone that occurred when using increased cutting clearances and the formation of cut-edge defects. The least unfavorable changes in the parameters of the hysteresis loop occurred for the experiments performed with cutting clearances of *h_c_* = 0.02 mm and *h_c_* = 0.04 mm and a cutting speed of *v* = 17.5 m/min. The highest maximum induction and the smallest coercivity then occurred. Reducing the cutting speed to *v* = 3 m/min with a clearance of *h_c_* = 0.06 mm resulted in a decrease in the value of saturation induction and an increase in coercivity. The induction of remanence also decreased. A further increase in the cutting speed value for this clearance value reduced the saturation induction, while the remanence induction and coercivity did not change significantly.

## 4. Optimization of the Process

On the basis of the obtained research results and the developed mathematical models of the process (regression function type II: for the magnetic hysteresis field *h_f_,* the height of the burr on the cut edge *h_z_* for cutting), an optimization task was developed. In industrial conditions, it is important to deliver products of planned, repeatable technological quality, and thus of functional quality. Selected features (*h_f_*, *h_z_*) determine both the construction quality (important, e.g., from the point of view of sheet-metal assembly) and electromagnetic quality (important, e.g., from the point of view of the efficiency of manufactured machines and electrical devices). The gradient method was used for multi-parameter optimization of the mechanical cutting process of electrical sheets. In industrial production, in addition to obtaining a high-quality product, the goal is to maximize efficiency. In the case under consideration, the efficiency of the mechanical cutting process was defined as W = f (*v*), where *v* is cutting speed.

The developed type II regression function for process optimization may be an objective function or a constraint function. The optimization task was defined as follows:


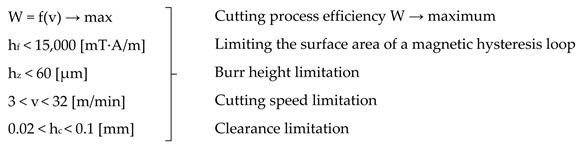


**Figure 17:**
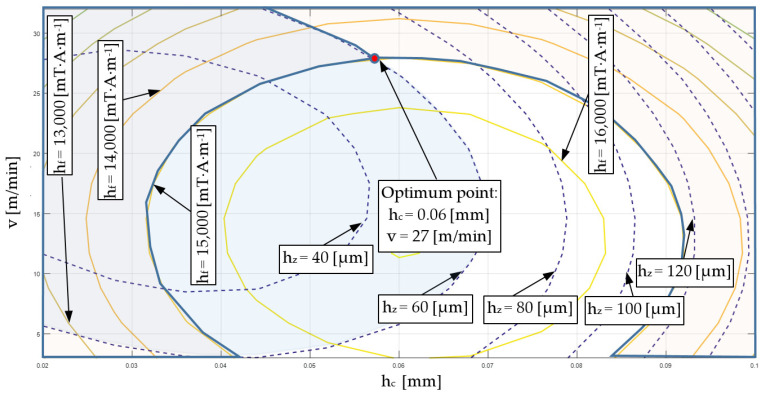
Graphical optimization of the cutting process.

The developed optimization task was solved using the graphical method (Figure 17). The optimal values of the process settings were determined as follows: optimal horizontal clearance, *h_c opt_*_._ = 0.06 mm; optimal cutting speed, *v_opt_* = 27 m/min. The designated settings guarantee high technological quality in the product with maximum efficiency in the process.

## 5. Practical Application of Analysis Results

**Figure 18:**
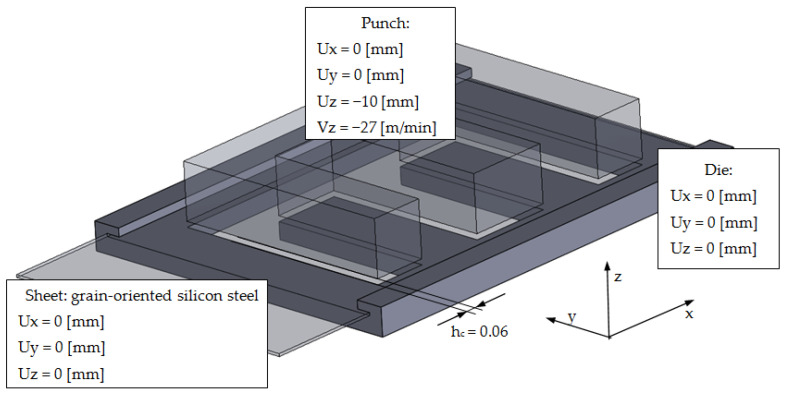
Geometrical model of blanking process with optimal technological parameters.

The conducted fragmentary experimental research, results analysis, and multi-criteria optimization of the cutting process enabled the development of an effective numerical model of the process for blanking parts for electric machines. Using the results, a computer model was developed with the finite element method for the process of blanking parts for the construction of an electrical transformer from grain-oriented electrical sheets. The developed model is aimed at verifying the results of fragmentary tests and implementing research conclusions into production. The industrial implementation of the research results will make it possible to obtain a polyoptimal product for mechanical (manufacturing) and electrical (efficiency of electrical devices) purposes. Figure 18 shows the FEM model of the sheet metal blanking process for the transformer core.

**Figure 19:**
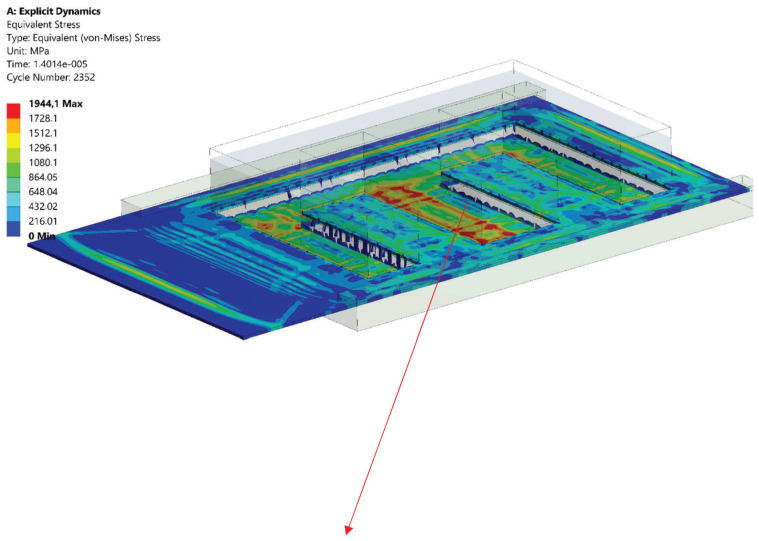

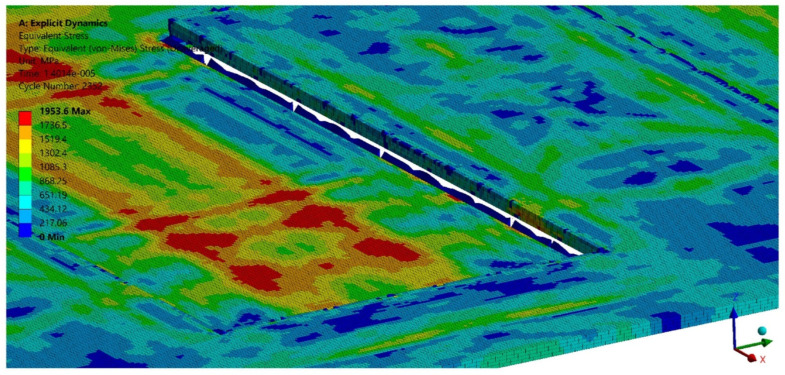
State of equivalent von Mises stress.

The solution of the developed equation for the motion of the object was found using the method of explicit integration (central difference) [51,52,53]. The results (state of reduced stresses) are shown in Figure 19.

**Figure 20:**
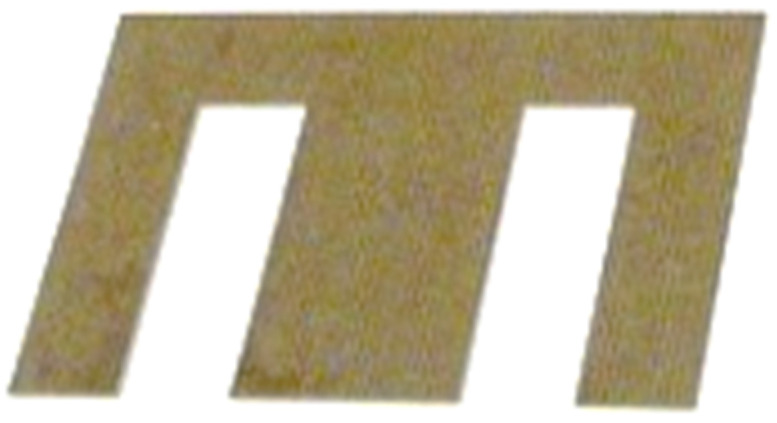
Transformer core sheet cut with optimal technological parameters from grain-oriented electrical steel.

The simulation results confirm the height of the burrs obtained in experimental tests, and thus the optimal machining parameters we determined for obtaining the correct product. The last stage of our study was the implementation of technological parameters in the manufacturing process. As a result, a polyoptimal product (mechanically and electrically) was obtained, shown in Figure 20. The product confirmed previous expectations and has been implemented for production.

## 6. Conclusions

The process of cutting electrical sheets is a very complex process. The technological quality of the product depends on many parameters related to both the condition of the workpiece and the conditions of the shearing process. The shear-slitting process is characterized by complex kinematics, which made the recommendations in the literature regarding blanking or punching processes unsuitable for use in this process. This explains the high burr formations, edge fracture, and rollover on cut surface that are observed in the shearing processes on production lines.

To enable a detailed analysis of these issues, an experimental analysis of the shear-slitting process was carried out, taking into account variable cutting conditions. As a result, the influence of the main control parameters on production lines on the quality of the cut edge and selected magnetic parameters of the material after the process was determined. Based on our results, the following conclusions can be formulated:

- Conducting tests for a material with an insulating coating, which has a composite structure, and for the same material without the coating, allowed for determining the local changes in the quality of the cut edge and identifying the appropriate cutting conditions for each of the cases. This creates new possibilities for production planning and the possibility of proper selection of machining parameters depending on whether the material will be cut with or without a coating.
- The analysis of the test results showed a strong influence of cutting speed and horizontal clearance on the formation of the width of individual zones on the cut surface, both in the case of sheets with and without an insulating coating. The test results indicate that the lowest process stability can be obtained at high cutting speeds above 24 m/min. This especially applies to variants of clearances above *h_c_* = 0.08 mm.
- The use of an insulating coating reduces the effect of cutting speed on the width of the sheared burnished zone (by approx. 15%). On the other hand, the effect of horizontal clearance increases (by approx. 20%). The coating increases the stability of the plastic flow process as well as the propagation and the course of cracking for clearances in the range of *h_c_* = 0.02–0.06 mm.
- In some cases, at the edge of the burr along the cut line, fragments of the insulating coating, which is a form of built-up edge on the cut surface, were observed. This can have a very negative effect on the magnetic properties and accelerate the wear of the cutting tools.
- As horizontal clearance increased, there was an excessive increase in the bending moment and local edge damage. The use of an insulating coating resulted in a narrowing of the ranges of favorable input parameters settings and an increase in roundness in the case of using minimal clearances and increased cutting speeds.
- The cutting process changes the shapes of the hysteresis loop in the areas of the upper curve of the characteristic and saturation. For reduced cutting clearances beyond the range of *h_c_* = 0.02–0.04 mm, the cutting speed mainly affected the characteristics of the saturation area and the maximum induction. In the case of increased cutting clearances, the value of the saturation induction decreased. This was especially visible in clearance values above *h_c_* = 0.08 mm. The increase in clearance also caused an increase in the intensity of coercivity and induction of remanence.
- Based on the research results and the use of graphical optimization, a set of acceptable solutions and an optimal solution were determined to ensure the highest quality of the cut edge and minimum disturbances in magnetic properties (*v* = 27 m/min, *h_c_* = 0.06 mm). The proposed approach enables the implementation of the process for other data.

## Figures and Tables

**Table 1:** Mechanical properties of non-coated ET 110-30LS steel (at *T* = 20 °C).

Density [kg/dm^3^]		R_p0.2_ [MPa]	R_m_ [MPa]	F_m_ [kN]	A_g_ [%]	Hardness [HB]	Hardness [HV]
7.8		318	332	1	11.4	148	157
$\bar{\sigma }$	1.1	10.88	9	0.03	2.79	2.98	2.06

**Table 2:** Mechanical properties of coated ET 110-30LS steel (at *T* = 20 °C).

Density [kg/dm^3^]		R_p0.2_ [MPa]	R_m_ [MPa]	F_m_ [kN]	A_g_ [%]	Hardness [HB]	Hardness [HV]
7.8		314	337	0.96	10.93	157	165
$\bar{\sigma }$	1.3	12.3	9.4	0.02	0.02	0.81	2.38

**Table 3:** The values of the studied factors.

Horizontal clearance, *h_c_*	0.02−0.1 mm
Vertical clearance, *c_v_*	0.1 mm
Slitting velocity, *v*	3−32 m/min
Rake angle, *α*	7°

## Data Availability

Data are available from authors upon request.
